# On the detectability of adjacency effects in ocean color remote sensing of mid-latitude coastal environments by SeaWiFS, MODIS-A, MERIS, OLCI, OLI and MSI

**DOI:** 10.1016/j.rse.2017.12.021

**Published:** 2018-05

**Authors:** Barbara Bulgarelli, Giuseppe Zibordi

**Affiliations:** European Commission, Joint Research Centre (JRC), Ispra, Italy

**Keywords:** Ocean color, Adjacency effects, SeaWiFS, MODIS, MERIS, OLCI, OLI, MSI

## Abstract

The detectability of adjacency effects (AE) in ocean color remote sensing by SeaWiFS, MODIS-A, MERIS, OLCI, OLI and MSI is theoretically assessed for typical observation conditions up to 36 km offshore (20 km for MSI). The methodology detailed in Bulgarelli et al. (2014) is applied to expand previous investigations to the wide range of terrestrial land covers and water types usually encountered in mid-latitude coastal environments. Simulations fully account for multiple scattering within a stratified atmosphere bounded by a non-uniform reflecting surface, sea surface roughness, sun position and off-nadir sensor view. A harmonized comparison of AE is ensured by adjusting the radiometric sensitivity of each sensor to the same input radiance. Results show that average AE in data from MODIS-A, and from MERIS and OLCI in reduced spatial resolution, are still above the sensor noise level (NL) at 36 km offshore, except for AE caused by green vegetation at the red wavelengths. Conversely, in data from the less sensitive SeaWiFS, OLI and MSI sensors, as well as from MERIS and OLCI in full spatial resolution, sole AE caused by highly reflecting land covers (such as snow, dry vegetation, white sand and concrete) are above the sensor NL throughout the transect, while AE originated from green vegetation and bare soil at visible wavelengths may become lower than NL at close distance from the coast. Such a distance increases with the radiometric resolution of the sensor. It is finally observed that AE are slightly sensitive to the water type only at the blue wavelengths. Notably, for an atmospheric correction scheme inferring the aerosol properties from NIR data, perturbations induced by AE at NIR and visible wavelengths might compensate each other. As a consequence, biases induced by AE on radiometric products (e.g., the water-leaving radiance) are not strictly correlated to the intensity of the reflectance of the nearby land.

## Highlights

• Quantification of adjacency effects for a wide range of coastal environments
• Comparison of adjacency effects for data from various ocean color sensors
• Relevance of adjacency effects as a function of the SNR of satellite sensors

## 1 Introduction and background

Quantitative optical remote sensing of seas and oceans started in 1978 with the launch of the NASA Coastal Zone Color Scanner (CZCS, 1978–1986). Among the ocean color sensors that followed, several ensured multi-annual observations: the NASA Sea-viewing Wide Field-of-view Sensor (SeaWiFS, 1997–2010), the NASA Moderate Resolution Imaging Spectroradiometer (MODIS-T, 1999-present on board Terra platform, and MODIS-A, 2002-present on board Aqua platform), the ESA Medium Resolution Imaging Spectrometer (MERIS, 2001–2012) and the most recent ESA Sentinel-3 Ocean and Land Color Instrument (OLCI, 2016-present). Optical remote sensing of the sea is also performed by sensors dedicated to land observations, such as the NASA Landsat-8 Operational Land Imager (OLI, 2013-present) and the ESA Sentinel-2 MultiSpectral Imagery (MSI, 2015-present). The latter acquires information of the sea only up to 20 km from the coast.

While the determination of the optical properties of the open ocean from satellite measurements is nowadays established, remote sensing of coastal waters still represents a challenge. In coastal areas the optical complexity of seawater adds to eventual perturbations from bottom and nearby land. Even more challenging are remote sensing observations of inland waters, for which contributions from the surrounding land cannot be altogether neglected. Nonetheless, apart from few focused investigations (Kiselev et al., 2015, Sterckx et al., 2015; Sei, 2015, Santer and Zagolski, 2009), standard processing techniques of ocean color data generally assume an optically homogenous underlying surface (Antoine and Morel, 1999, Gordon and Wang, 1994), thus neglecting possible top-of-atmosphere (TOA) radiance contamination between neighboring surfaces with different reflectance. The latter phenomenon is usually termed *adjacency effects* (AE) and can be quantified through the *adjacency radiance L*_*adj*_, which defines the difference in the TOA radiance between the case accounting for the nonuniformity of the underlying reflecting surface and the case assuming a uniform surface. As such, it can vary from positive to negative values.

A methodology was recently developed to accurately simulate AE in ocean color data from coastal areas (Bulgarelli et al., 2014). Such a methodology relies on three-dimensional (3D) MonteCarlo (MC) radiative transfer simulations fully accounting for multiple scattering within a stratified atmosphere bounded by a non-uniform and anisotropic reflecting surface, sun position, off-nadir sun-sensor geometries, wind-induced sea surface roughness, and coastal morphology. Its accuracy was evaluated through comparisons with data from an alternative and extensively benchmarked numerical code based on the finite element method (FEM) (Bulgarelli et al., 1999) and with data from the literature (Sei, 2007). The methodology was applied to quantify AE at a specific test site, namely, the region of the northern Adriatic sea hosting the Aqua Alta Oceanographic Tower (AAOT, 45.31°N, 12.51°E), a validation site included in the Ocean Color component of the Aerosol Robotic Network (AERONET-OC) (Zibordi et al., 2009b) and where comprehensive bio-optical in situ measurements are collected since 1995 (Zibordi et al., 2002). The investigation also included a sensitivity analysis on commonly applied approximations (e.g., single scattering, Lambertian reflectance of the sea, nadir observation, straight coastline). For the same test site, subsequent studies addressed the biases induced by AE in radiometric products (Bulgarelli et al., 2017) and the use of quantified AE to reduce annual and intra-annual overall biases (Bulgarelli et al., in preparation).

Although the above results allow drawing general considerations on AE, quantitative estimates are still representative of the sole observation conditions typical of mid-latitude coastal regions characterized by a cropland ecosystem and Case-1 to Case-2 moderately turbid waters. Consequently, their validity cannot be generically extended to any observation condition and any coastal environment. This holds even truer for simulated AE in the right proximity of the coast, heavily influenced by the peculiar morphology of the Venice Lagoon.

A more comprehensive picture of adjacency perturbations in coastal data definitely requires the evaluation of the effects in a wider combination of coastal environments and observation conditions, efficiently achievable through theoretical simulations. Indeed, direct estimates of AE from at-sensor satellite measurements are confined to limited observational conditions (e.g., a negligible water contribution (Feng and Hu, 2017)), while the direct evaluation of AE in satellite-derived products might be hindered by mechanisms of compensation within the applied atmospheric correction (AC) scheme. It is recalled that an attempt to estimate AE in SeaWiFS products (i.e., the aerosol optical thickness at 865 nm, and the normalized water-leaving radiance at 670 nm) derived with the SeaWiFS Data Analysis System (SeaDAS) (Franz et al., 2007, Ahmad et al., 2010) along transects starting from the coast and intercepting selected AERONET-OC sites, did not provide any firm evidence of appreciable AE (Zibordi et al., 2009a), further explained as the consequence of compensations triggered within the SeaDAS algorithm (Bulgarelli et al., 2017).

The need to extend the set of AE simulations with respect to previous studies (e.g., Bulgarelli et al., 2014, Bulgarelli et al., 2017) finds additional justification in a recent publication by Feng and Hu (2017) indicating differences in the immediate vicinity of the coast between AE empirically estimated from MODIS-A near-infrared (NIR) data of Madagascar coastal waters and AE simulated by Bulgarelli et al. (2014) in the northern Adriatic Sea. The present work suggests that those differences might be explained by uncertainties affecting the determination of AE across different methods at very short distances from the coast, and above all, by the diverse observation conditions (i.e., land albedo, coastal morphology, solar illumination, aerosol load and scale height).

The specific aim of the present manuscript is to extend the theoretical quantification of AE to a large set of test cases representative of a wide range of typical observation conditions for mid-latitude coastal environments.

In specific, the methodology developed in Bulgarelli et al. (2014) is applied to simulate AE at visible and NIR wavelengths for typical atmospheric and sun-sensor geometries along a study transect extending perpendicular to a half-plane of uniform and isotropic land albedo, and whose coastline is oriented in the South-North direction. The optical properties of the water are assumed to be constant along the study transect and described by spectra of the remote sensing reflectance *R*_*rs*_ typical of European waters (Eastern Mediterranean, Ligurian Sea, northern Adriatic Sea, Western Black Sea, English Channel, and Baltic Sea) (Zibordi et al., 2011), while the bi-directional reflectance of the wind-roughed sea surface is modeled according to Kisselev and Bulgarelli (2004). For the land cover, reflectance spectra of most common terrestrial environments (grass, dry grass, deciduous trees, conifers, concrete, snow of different grain size, white and brown sand, brown and pale brown loam) have been obtained from the comprehensive Advanced Spaceborne Thermal Emission and Reflection Radiometer (ASTER) spectral library (Baldridge et al., 2009).

Results are analyzed with regards to the radiometric sensitivity of SeaWiFS, MODIS-A, MERIS, OLCI, OLI and MSI, where the harmonized comparison of AE among different sensors is ensured by adjusting the radiometric sensitivity of OLCI, OLI and MSI to the same input radiance utilized by Hu et al. (2012) to standardize the signal-to-noise ratio (SNR) of SeaWiFS, MODIS-A and MERIS.

## 2 Methods

### 2.1 Simulation procedure

The methodology described in Bulgarelli et al. (2014) has been adopted to estimate percent adjacency contributions at the sensor level *ξ*_*L*_*tot*__ = *L*_*adj*_/*L*_*tot*_ · 100, where *L*_*tot*_ is the TOA signal, and the adjacency radiance *L*_*adj*_ is modeled as:

(1) $$L_{\mathrm{adj}}=L_{l}^{\mathrm{TOA}}-{\tilde{L}}_{w}^{\mathrm{TOA}}-{\tilde{L}}_{\mathrm{ss}}^{\mathrm{TOA}},$$

with *L*_*l*_^*TOA*^ representing the land contribution (i.e., the radiance at the sensor originating from the area covered by land), and ${\tilde{L}}_{w}^{\mathrm{TOA}}$ and ${\tilde{L}}_{\mathrm{ss}}^{\mathrm{TOA}}$ indicating the masked water and the masked sea surface contributions, respectively (i.e., the water-leaving radiance and the sea surface radiance that would reach the sensor from the same area if still covered by the sea). It is mentioned that the term ${\tilde{L}}_{\mathrm{ss}}^{\mathrm{TOA}}$ is sometimes called the *Fresnel mask* (Santer and Schmechtig, 2000). Eq. (1) can be further parameterized as (see Bulgarelli et al., 2014 for details):

(2) $$L_{\mathrm{adj}}=\left\{\rho_{l}/\left[\pi \left(1-\rho_{l}S\right)\right]-R_{\mathrm{rs}}/\left(1-\rho_{\mathrm{sea}}S\right)\right\}\cdot C-W.$$

where *ρ*_*l*_ represents the albedo of the land assumed isotropic and spatially homogeneous; *S* is the atmospheric spherical albedo of the bottom of the atmosphere; *ρ*_*sea*_ is the albedo of the sea (Mobley, 1994); and the expressions for functions *C* and *W* are given in Bulgarelli et al. (2014). The functions *C* and *W* depend on the illumination and observation geometry, on the land/sea spatial extension, as well as on the atmospheric optical properties. The described approach allows decoupling the land and water optical properties from atmospheric scattering, yet fully accounting for sea surface roughness. It is pointed out that the term 1/(1 − *ρ*_*sea*_ *S*) in Eq. (2) is exclusively used in the determination of ${\tilde{L}}_{w}^{\mathrm{TOA}}$ to account for multiple reflections by the sea surface of atmospherically scattered light, whereas the bi-directional reflectance properties of a wind-roughed sea surface are accurately accounted for in the computation of ${\tilde{L}}_{\mathrm{ss}}^{\mathrm{TOA}}$. While the simulation of *C* and *W* requires a full 3D description of the propagating system, the simulation of *S* can be performed with a plane-parallel radiative transfer code, while the input parameters *ρ*_*l*_ and *R*_*rs*_ can be extrapolated from satellite-derived or in situ data. Therefore, once functions *C* and *W* are computed for a given geometric and atmospheric case, the proposed modeling allows a straightforward evaluation of AE for a wide variety of land and water spectral signatures.

The 3D Novel Adjacency Perturbation Simulator for Coastal Areas (NAUSICAA) MC code (Bulgarelli et al., 2014), whose accuracy was set to meet average SeaWiFS, MODIS and MERIS radiometric sensitivities, has been utilized to perform the simulations of functions *C* and *W*. The FEM plane-parallel numerical code (Bulgarelli et al., 1999), already extensively benchmarked with other popular radiative transfer codes (Bulgarelli et al., 1999, Bulgarelli and Doyle, 2004, Bulgarelli et al., 2003) and additionally used to perform radiative transfer simulations in realistic cases (Bulgarelli et al., 2003, Bulgarelli and Mélin, 2003, Bulgarelli and Zibordi, 2003, Zibordi and Bulgarelli, 2007), has also been utilized to simulate the atmospheric radiance and its components. Simulations have been carried out at the SeaWiFS-equivalent center-wavelengths i.e., for λ = 412, 443, 490, 510, 555, 670, 765 and 865 nm.

### 2.2 Selected test cases

**Fig. 1:**
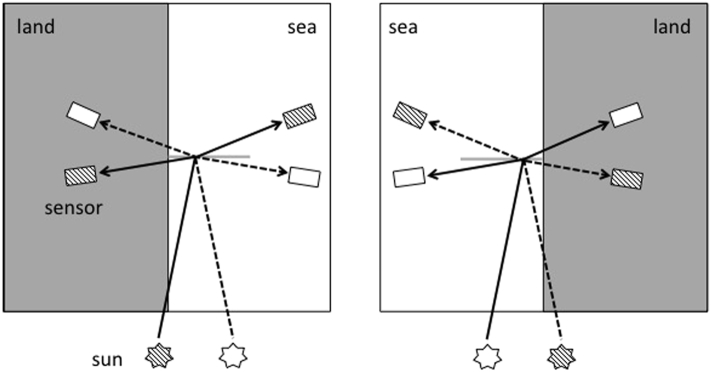
Geometry of illumination and observation adopted in the simulations. The gray horizontal line represents the study-transect.

Simulations have been performed for typical mid-latitude observation conditions (identified by a sun zenith angle θ_0_ = 45°, Ångström coefficient α = 0.05, Ångström exponent *ν* = 1.7, aerosol scale height H_*a*_ = 1.2 km) assuming a stratified atmosphere resolving the vertical distribution of gas molecules and aerosol (Bulgarelli et al., 2014). Simulated values are provided from 1 to 36 km off the coast (at 2 km increments) along a study transect extending perpendicularly to a half-plane of uniform and isotropic land albedo, and whose coastline is oriented in the South-North direction (Fig. 1). Both cases of a land located in the western and eastern half-planes have been considered. It is noted that a previous analysis evidenced the feasibility to neglect the actual coastal morphology without any loss in accuracy, apart for enclosed basins (Bulgarelli et al., 2014).

The same set of parameters utilized to describe solar and sensor geometries for SeaWiFS, MODIS-T, MODIS-A and MERIS observations in the northern Adriatic Sea (Bulgarelli et al., 2014) has been considered suitable to describe observation conditions for OLCI, OLI and MSI (Table 1 and Fig. 1). Indeed, the equatorial crossing time (ECT) of OLCI and OLI is the same as MERIS (10:00 Mean Local Solar (MLS) time in descending node), while the ECT of MSI (10:30 MLS time in descending node) coincides with that of MODIS-T. As such, the same illumination geometry applies. For OLCI, all viewing angles θ_v_ = 5°, 20° and 50° are suitable. Conversely, for MSI (whose field-of-view (FOV) is 20.6°) only θ_v_ = 5° and 20° apply, while for OLI (whose FOV is 15°) θ_v_ = 5° is the sole appropriate.

**Table 1:**
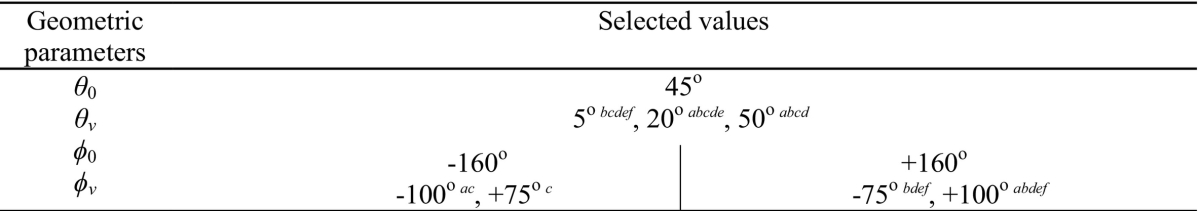
Parameters defining the illumination and observation geometries adopted in the simulations for each sea/land configuration (see Fig. 1).

^*a*^SeaWiFS, ^*b*^MERIS, ^*c*^MODIS-A, ^*d*^OLCI, ^*e*^OLI, ^*f*^MSI. θ-angles are determined with respect to the local vertical; ϕ-angles are counted clock-wise from the north direction (as generally adopted in satellite geolocation).

A large set of optical properties for the two half-planes covered by uniform land and water, have been selected. Adjacency contributions have been quantified for a total of 12 × 6 land-water cases (see 2.2.1, 2.2.2). For each test case, all suitable observation geometries (Table 1 and Fig. 1) for both sea/land configurations and all reference wavelengths have been considered.

#### 2.2.1 Land surface optical properties

**Fig. 2:**
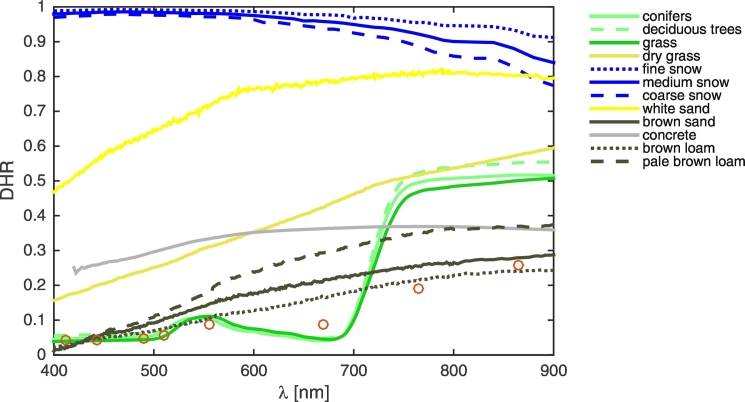
Values of DHR for selected land covers. Empty dots represent the land albedo utilized to simulate AE in the northern Adriatic Sea (Bulgarelli et al., 2014), as inferred from the MODIS climatological database (Moody et al., 2008).

Spectral *directional-hemispherical reflectances* (DHR, i.e., the reflectance for incoming light from a single direction (Martonchik et al., 2000)) for grass, dry grass, deciduous trees, conifers, concrete, snow of different grain size, white and brown sand, brown and pale brown loam, have been extracted from the ASTER spectral library (Baldridge et al., 2009) and have been assumed to approximate the land albedo *ρ*_*l*_ (see Fig. 2). For comparison, Fig. 2 also indicates the values of *ρ*_*l*_ utilized for the simulation of AE in the northern Adriatic Sea (Bulgarelli et al., 2014), as inferred from the MODIS climatological database (Moody et al., 2008).

As illustrated in Fig. 2, the largest DHR values occur for snow, with little dependence on wavelength and grain size. DHRs of green vegetation typically display low values in the visible and large values in the NIR, with small dissimilarities among the different selected vegetation types. Conversely, dry grass exhibits a significant increase in reflectance at shorter wavelengths. White sand is characterized by a large reflectance, which is comparable to that of snow in the NIR, while brown sand, brown loam and pale brown loam soils (representative of the most diffuse soil types in European coastal regions (USDA, 2005)) show a smooth monotonic increase of reflectance from blue to NIR wavelengths. Finally, concrete is characterized by a rather flat spectrum with DHR of approximately 0.3.

Although the present selection of land covers is not exhaustive, their combination can be considered representative of the ecosystems listed in the IGBP Land Ecosystem Classification Map Image (IGBP, 2000). It is indeed clear that the actual surface is a composite of different land covers. For example, the cropland ecosystem can be modeled as a weighted composite of vegetation and soil, which varies according to cropland type, moisture content and phenological state, and whose reflectance is closer to that of vegetation in summer and to that of soil in winter.

#### 2.2.2 Water optical properties

Typical spectral values of the remote sensing reflectance *R*_*rs*_ have been extracted from the comprehensive Bio-Optical mapping of Marine Properties (BiOMaP) dataset (Zibordi et al., 2011). It is mentioned that the BiOMaP program was established in 2004 to provide cross-site consistent inherent (IOPs) and apparent (AOPs) optical properties of the European seas in support of ocean color applications. The sampled marine regions embrace a wide set of bio-optical conditions (see Berthon et al., 2008, and references therein) ranging from Case-1 waters, whose bio-optical properties are well correlated to the Chlorophyll concentration *Chl* (Loisel and Morel, 1998), to a number of complex Case-2 waters, whose bio-optical properties derive by the simultaneous presence of not-covarying optically significant components, like *Chl*, color dissolved organic matter (CDOM) and suspended sediments (D'Alimonte et al., 2007). Specifically, spectral average values of *R*_*rs*_ measured in the Eastern Mediterranean (EMED, including areas of the southern Adriatic Sea), the Ligurian Sea (LIGS), the northern Adriatic Sea (NADR), the western Black Sea (BLKS), the English Channel (ECHN), and the Baltic Sea (BLTS) have been considered. In this study *R*_*rs*_ at NIR wavelengths has been assumed negligible. The latter choice is justified by noting that the reflectance of the land in the NIR is much larger than the reflectance of the sea, and by recalling that a negligible difference in the simulation of the adjacency radiance in NADR waters was observed when assuming a non-null NIR water-leaving radiance. This was quantified as the 0.5 and 0.9 quantiles of the values determined at the AAOT with SeaDAS from a set of 1124 SeaWiFS data (Bulgarelli et al., 2017).

**Fig. 3:**
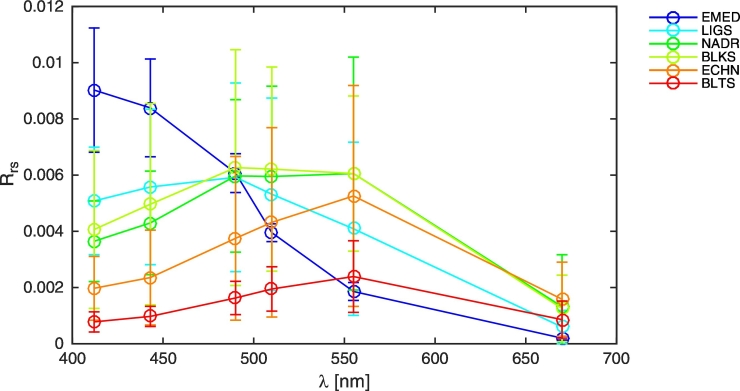
Spectral values of *R*_*rs*_ [sr^− 1^] for selected waters. Error bars indicate standard deviations.

The spectral plots of mean *R*_*rs*_ for the considered water types are given in Fig. 3. EMED and LIGS waters represent oligotrophic and mesotrophic Case-1 waters, respectively; NADR and ECHN are instead characterized by Case-2 waters moderately dominated by detritus or mineral particles from rivers discharge or tidal resuspension; BLKS waters occasionally offer unique concentrations of coccolithophores in the central-western basins and of sediments in proximity of the Danube sea; the BLTS are Case-2 CDOM-dominated waters with high concentration of dissolved humic matter and varying concentration of detritus particles in its different sub-basins (Berthon and Zibordi, 2010). Summary of average IOPs for the selected BiOMaP regions can be found in Zibordi et al. (2011).

#### 2.2.3 Modeling of the sea surface

The sea surface has been modeled as roughed by a wind speed of 3.3 ms^− 1^, typically recorded at the AAOT (Berthon et al., 2002). Notably, simulations of AE in the northern Adriatic Sea did not show sensitivity to wind speed variation within 1 and 6 ms^− 1^ (Bulgarelli et al., 2017). The expression of the directional reflectance properties of a wind-generated rough sea surface, applicable for all reflection angles, has been taken from Kisselev and Bulgarelli (2004). In fact, the well-known expression from Cox and Munk (1954) tends to infinity for angles of reflection close to the horizontal direction, and is hence less suitable for implementation in numerical methods to solve of the radiative transfer equation.

### 2.3 Sensors radiometric sensitivity

Higher radiometric sensitivity allows resolving smaller changes of the target signal, but it also implies higher sensitivity to spurious perturbations such as those generated by nearby land. A critical parameter describing the radiometric resolution of a sensor is the signal-to-noise ratio, SNR. The comparison of AE among different sensors requires the SNR to be defined and computed in a harmonized way, i.e., for the same input signal. Nonetheless the radiometric sensitivity of each sensor is unfortunately specified for different radiance values. To overcome this, Hu et al. (2012) carried out a re-quantification of the SNR of SeaWiFS, MODIS-A, and MERIS under uniform conditions. A typical at-sensor spectral radiance *L*_*typ*_ was determined from MODIS-A cloud-free ocean scenes and the SNR of SeaWiFS, MODIS-A and MERIS was directly estimated from data characterized by TOA radiances close to *L*_*typ*_. For comparison, pre-launch SNRs of SeaWiFS and MODIS-A from sensors specifications were also adjusted to the same input radiance *L*_*typ*_ assuming proportionality between the SNR and the square root of the input signal. It is indeed recalled that in modern radiometers, detector and readout noise are dominated by the shot (or photon) noise that describes the variation in the number of photons *N*_*p*_ detected by the sensor per unit time. Since the latter follows a Poisson distribution, whose standard deviation is the square root of *N*_*p*_, the overall noise can be assumed proportional to the square root of the input radiance (Moses et al., 2012). This scheme has been here utilized to scale the latest on-orbit performance SNR of OLCI, OLI and MSI (specified at different reference input radiances *L*_*ref*_) to the same input radiance *L*_*typ*_ (see *SNR*@*L*_*typ*_ values listed in Table 2, Table 3, Table 4, respectively). Results from Hu et al. (2012) suggest that until the input reference radiance is within ± 50% of *L*_*typ*_, uncertainties on *SNR*@*L*_*typ*_ are generally lower than 10%. For the aim of the present study it should hence be considered that uncertainties on *SNR*@*L*_*typ*_ might exceed 10% at all bands for the MSI sensor and at the red and infrared bands for OLI. Furthermore, considering that at these bands *L*_*ref*_ is larger than *L*_*typ*_, the computed *SNR*@*L*_*typ*_ are likely to underestimate the actual values (Hu et al., 2012).

**Table 2:** SNR for OLCI-RR at center-wavelengths *λ* characterized by bandwidth Δλ after adjustment *to L*_*typ*_ (*SNR@L*_*typ*_). SNR at *L*_*ref*_ [Wm^− 2^ μm^− 1^ sr^− 1^] from sensor specifications (*SNR@L*_*ref*_) are also indicated (S3 MPC 02/2017). It is mentioned that OLCI-RR measurements are performed with spatial resolution Δs = 1200 m at all bands. SNR of OLCI-FR (Δs = 300 m) are ¼ of those of OLCI-RR. The SeaWiFS-equivalent center-wavelengths utilized in the study are indicated in bold.

λ	Δλ	*SNR@L*_*typ*_	*SNR@L*_*ref*_	*L*_*ref*_
400	15	2736.4	2356	62.95
**412.5**	**10**	**2488.7**	**2388**	**74.14**
**442.5**	**10**	**2254.5**	**2183**	**65.61**
**490**	**10**	**2006.0**	**2000**	**51.21**
**510**	**10**	**1969.6**	**1985**	**44.39**
**560**	**10**	**1686.0**	**1798**	**31.49**
620	10	1481.0	1607	21.14
**665**	**10**	**1373.3**	**1553**	**16.38**
673.75	7.5	1179.0	1337	15.70
681.25	7.5	1166.6	1326	15.11
708.75	10	1260.0	1424	12.73
753.75	7.5	959.5	1128	10.33
761.25	2.5	541.4	502	6.09
764.375	3.75	656.2	663	7.13
767.5	2.5	532.8	559	7.58
**778.75**	**15**	**1276.1**	**1513**	**9.18**
**865**	**20**	**1004.2**	**1238**	**6.17**
885	10	665.4	819	6.00
900	10	619.0	688	4.73
940	20	643.2	533	2.39
1020	40	293.7	346	3.86

**Table 3:** *SNR@L*_*typ*_ for OLI (see Table 2). Values of *SNR@L*_*ref*_ are from Morfitt et al. (2015). Center-wavelength, bandwidth and spatial resolution are indicated by λ, Δλ and Δs, respectively. The SeaWiFS-equivalent center-wavelengths utilized in the study are indicated in bold.

λ	Δλ	Δs	*SNR@L*_*typ*_	*SNR@L*_*ref*_	*L*_*ref*_
**443**	**20**	**30**	**306.5**	**237**	**40**
**482**	**65**	**30**	**417.5**	**367**	**40**
**561**	**75**	**30**	**283.5**	**304**	**30**
590	180	15	145.4	148	23
**655**	**50**	**30**	**173.0**	**227**	**22**
**865**	**40**	**30**	**107.2**	**201**	**14**
1373	30	30	54.4	160	6
1609	100	30	77.5	267	4
2201	200	30	54.0	327	1.7

**Table 4:** *SNR@L*_*typ*_ for MSI (see Table 2). Values of SNR@L_ref_ are from S2 MPC (03/2017). Center-wavelength, bandwidth and spatial resolution are indicated by λ, Δλ and Δs, respectively. The SeaWiFS-equivalent center-wavelengths utilized in the study are indicated in bold.

λ	Δλ	Δs	*SNR@L*_*typ*_	*SNR@L*_*ref*_	*L*_*ref*_
**443**	**20**	**60**	**1009.2**	**1372**	**129**
**490**	**65**	**10**	**135.8**	**214**	**128**
**560**	**35**	**10**	**115.8**	**249**	**128**
**665**	**30**	**10**	**79.2**	**230**	**108**
705	15	20	93.6	253	74.5
**740**	**15**	**20**	**75.5**	**220**	**68**
783	20	20	70.1	227	67
842	115	10	46.4	221	103
**865**	**20**	**20**	**44.8**	**161**	**52.5**
945	20	60	137.2	222	9
1375	30	60	130.8	390	6
1610	90	20	47.1	159	4
2190	180	20	40.3	217	1.5

**Fig. 4:**
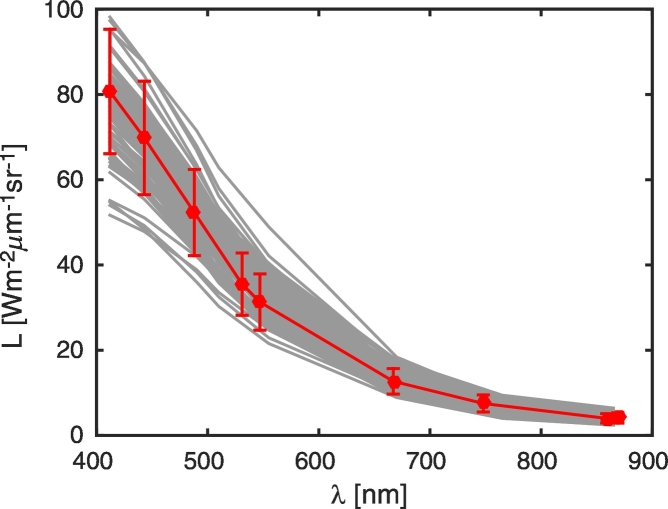
Filled circles indicate the spectral values *L*_*typ*_ and their standard deviations at MODIS-A center-wavelengths as provided by Hu et al. (2012). Gray lines indicate TOA radiances *L*_*tot*_ from a set of cloud-free SeaWiFS sample images acquired at the AAOT between 2002 and 2008.

Remarkably, sample cloud-free SeaWiFS images acquired at the AAOT between 2002 and 2008 and suitable for match-ups with in situ data show TOA radiances in optimal agreement with *L*_*typ*_ provided by Hu et al. (2012) (see values displayed in Fig. 4). This confirms the suitability of the recomputed SNRs for satellite observations of mid-latitude coastal waters.

**Fig. 5:**
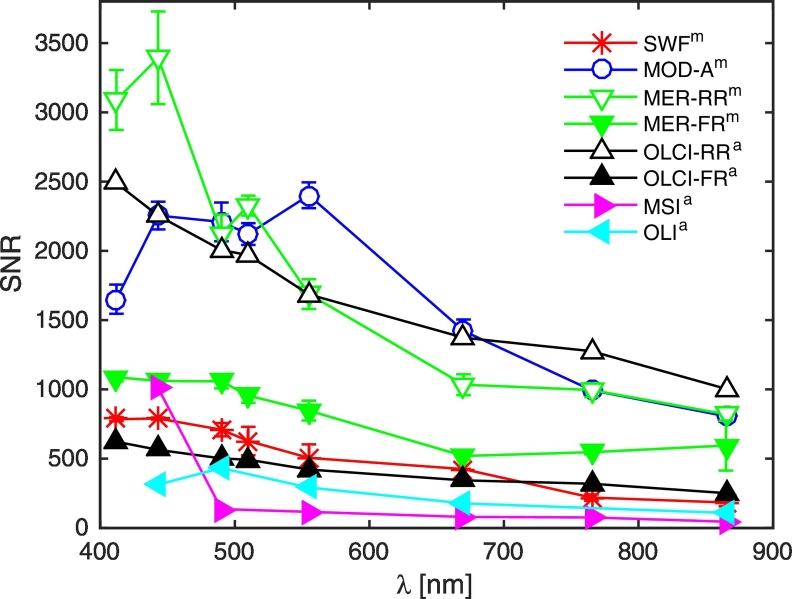
Spectral values of SNR at SeaWiFS-equivalent center-wavelengths for the various sensors. Suffix ‘m’ refers to SNR values and standard deviations provided by Hu et al. (2012). Suffix ‘a’ refers to on-orbit performance SNR after adjustment to the same input radiance *L*_*typ*_(*SNR@L*_*typ*_ in Table 2).

The harmonized SNR values for the considered sensors are spectrally represented in Fig. 5 at the SeaWiFS-equivalent center-wavelengths. Selected center-wavelengths for OLCI, OLI and MSI are highlighted in Table 2, Table 3, Table 4. MODIS-A selected center-wavelengths are those of the ocean color bands at 1 km spatial resolution. MERIS selected center-wavelengths coincide to those chosen for OLCI. It is remarked that the OLCI and MERIS band at 778 nm has been chosen among the other bands close to 765 nm because it is utilized to perform the atmospheric corrections (Antoine and Morel, 1999). Notably, OLCI and MERIS acquire data in full (0.3 km, FR) and reduced (1.2 km, RR) spatial resolution with a significantly different SNR. The highest radiometric sensitivity characterizes MODIS-A, MERIS-RR and OLCI-RR. In specific, MERIS-RR is the most sensitive sensor at blue-green wavelengths, MODIS-A around 555 nm, OLCI-RR at longer wavelengths. The radiometric sensitivity of MERIS-FR is slightly larger than that of SeaWiFS, while the radiometric performance of OLCI-FR (equal to ¼ of that of OLCI-RR) is slightly lower. As expected, the lowest SNR values are found for the MSI and OLI sensors that were developed for land applications. Indeed, land observations require a high spatial resolution to capture the large spatial variability of land optical properties and a high radiometric range to accommodate signals from bright targets. Conversely, they do not need the high radiometric resolution required by ocean color sensors to discriminate small variations of the water signal. Notably, the SNR of the MSI band at 443 nm (characterized by the coarsest spatial resolution) is significantly high, and close to that of MERIS-FR. At other center-wavelengths the SNR of MSI becomes instead the lowest. It is mentioned that MODIS-A land bands (with 250–500 m spatial resolution), also used for ocean color applications, are characterized by SNR values laying in between those of OLI and MSI.

**Fig. 6:**
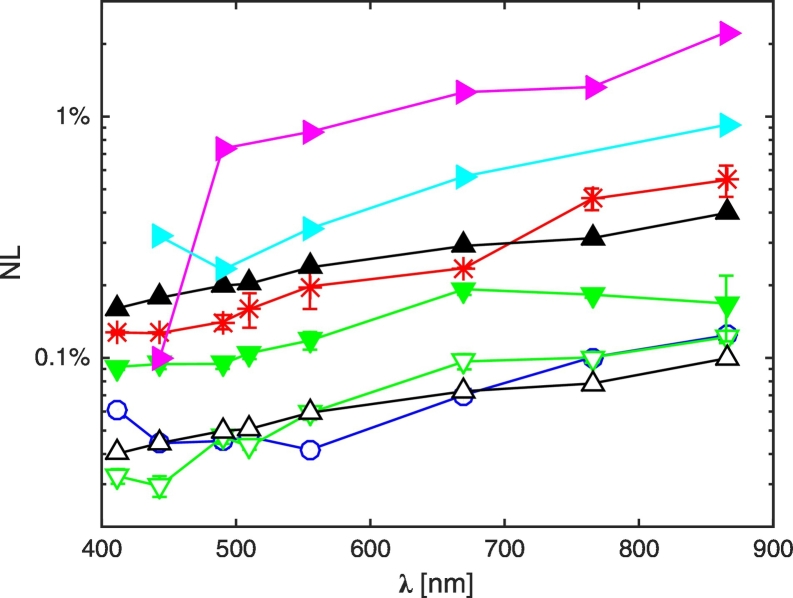
Spectral values of NL at SeaWiFS-equivalent center-wavelengths for the various sensors. Symbols are as in Fig. 5.

In the following of the paper the percent noise level (NL) values (i.e., NL = 1/SNR·100) displayed in Fig. 6 are utilized for comparison with *ξ*_*L*_*tot*__. Any adjacency radiance contribution lower than NL is regarded as not discriminable from noise, i.e., not detectable.

## 3 Results and discussion

Adjacency contributions *ξ*_*L*_*tot*__ for simulations along the study transect are presented in Section 3.1 with specific focus on the detectability of AE in satellite data from each considered sensor. Section 3.2 illustrates benchmarks with independent data. An analysis of biases induced by AE on satellite radiometric products is finally presented in Section 3.3.

### 3.1 Adjacency contributions at the sensor

**Fig. 7:**
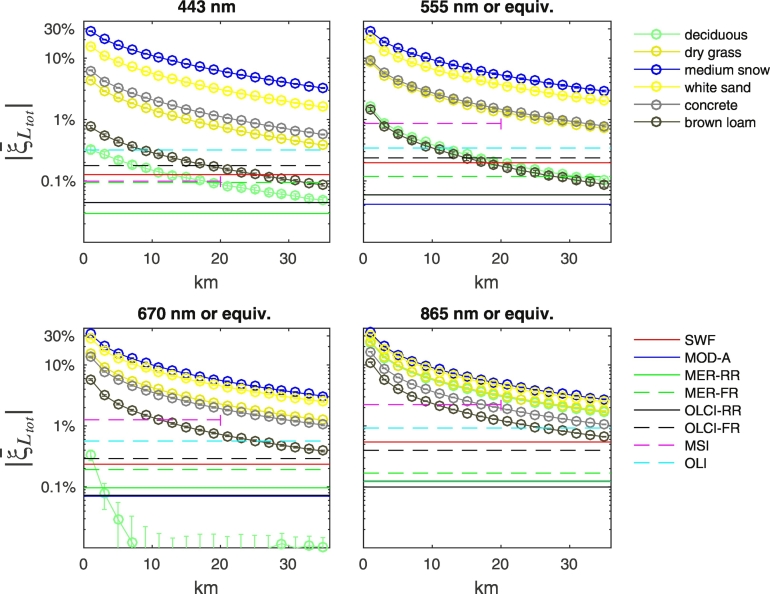
Values of $\left|{\bar{\xi }}_{L_{\mathrm{tot}}}\right|$ at representative center-wavelengths along the study transect as a function of the distance from the coast for NADR Case-2 moderately sediment-dominated waters and different land covers. Error bars represent uncertainties computed assuming uncorrelated contributions. Horizontal lines indicate the spectral NL values for the various sensors.

The absolute value of mean adjacency contributions ${\bar{\xi }}_{L_{\mathrm{tot}}}$ (for 24 observation geometries obtained by combining parameters detailed in Table 1) is summarized in Fig. 7 together with sensors NL. Results are presented at sample wavelengths for NADR waters and representative land covers. Data for fine and coarse snow are neither presented nor discussed being equivalent to those of medium grain snow. Data for conifers and grass, brown sand, and pale brown loam have been omitted being similar to those of deciduous trees and brown loam, respectively.

As expected, values of $\left|{\bar{\xi }}_{L_{\mathrm{tot}}}\right|$ monotonically decrease with the distance from the coast and their magnitude increases with the spectral albedo of the land cover. For all sensors, mean adjacency contributions in the presence of snow, white sand, concrete and dry vegetation, are above the spectral NL values throughout the study-transect and at all wavelengths. Conversely, in the presence of bare soil and green vegetation, adjacency contributions might become lower than NL within the transect at a distance that increases with the radiometric sensitivity of the sensor. As an example, average perturbations for brown loam at 443 nm become lower than NL at ~ 9 km offshore for OLI, ~ 19 km for OLCI-FR, ~ 25 km for SeaWiFS, ~ 33 km for MERIS-FR, while they are above the noise level throughout the whole 20 km-transect for MSI and at 36 km offshore for MODIS-A, OLCI-RR and MERIS-RR. It is remarked that mean adjacency contributions drop below the noise level at distance from the coast shorter than 36 km (20 for MSI): i) at the sole red center-wavelengths with green vegetation for the highly sensitive MODIS-A, MERIS-RR and OLCI-RR; ii) at all visible center-wavelengths for SeaWiFS, OLCI-FR and MERIS-FR, OLI and MSI; iii) at additional NIR center-wavelengths for MSI and OLI measurements in the presence of bare soil. It is mentioned that adjacency contributions at the blue wavelengths for green vegetation and bare soil are negative.

**Fig. 8:**
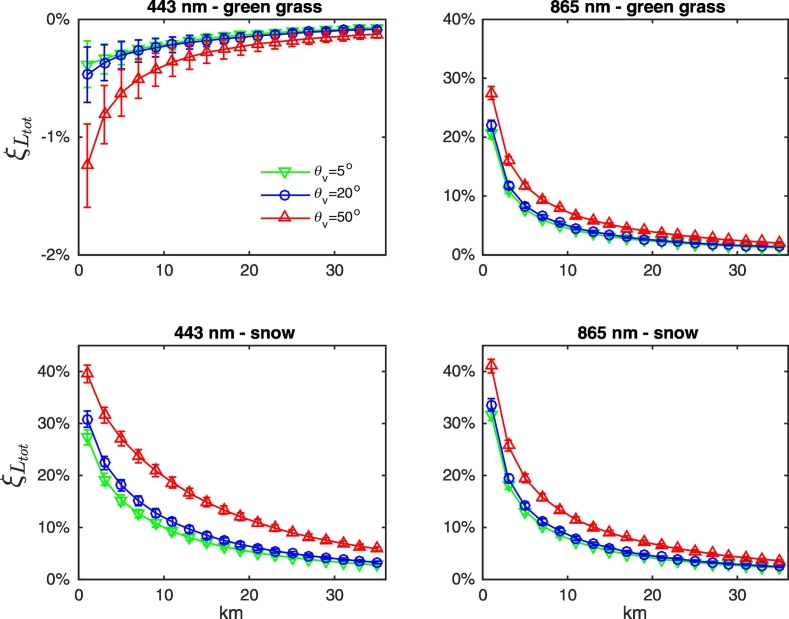
Values of *ξ*_*L*_*tot*__ at representative center-wavelengths along the study transect as a function of the distance from the coast for NADR waters in the presence of (upper panels) green grass and (lower panels) snow. Observations are for ϕ_0_ = 160°, ϕ_v_ = − 75° and land located in the western half-plane. Error bars represent uncertainties. (For interpretation of the references to color in this figure legend, the reader is referred to the web version of this article.)

**Fig. 9:**
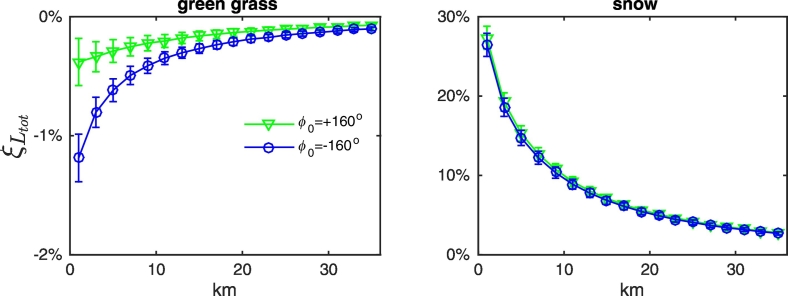
Values of *ξ*_*L*_*tot*__ at 443 nm along the study transect as a function of the distance from the coast for NADR waters in the presence of (left panel) green grass and (right panel) snow. Observations are from over the land (located in the western half-plane) and for θ_v_ = 5°. Error bars represent uncertainties. (For interpretation of the references to color in this figure legend, the reader is referred to the web version of this article.)

Different observation geometries, as well as the mutual location of sun, sensor and land, influence the actual adjacency contributions. In particular, results confirm a general increase of AE with the viewing angle (see Fig. 8 for green grass and snow at 443 and 865 nm). Adjacency perturbations also show a dependence on the position of the sun with respect to the land (see Fig. 9). Indeed, due to the anisotropy of the sea surface, the value of term *W* (the *Fresnel mask*) in Eq. (2) increases with the portion of land laying in the solar half-plane. This dependence is clearly more significant when land and sea albedos are closer (as for green vegetation and bare soil at blue wavelengths), because the contribution of *W* to *L*_*adj*_ is more important. Under these same conditions, results are expected to be more sensitive to actual roughness of the sea surface. Conversely, when the land reflectance is consistently larger than the sea one (i.e., throughout the spectrum for snow, dry vegetation, white sand and concrete, or at the sole NIR wavelengths for green vegetation and bare soil), AE become sensitive to the position of the sensor with respect to the land (see Fig. 9). This is mainly relevant for slanted observations, for which AE appear consistently larger when the sensor is observing the sea from over the land.

**Fig. 10:**
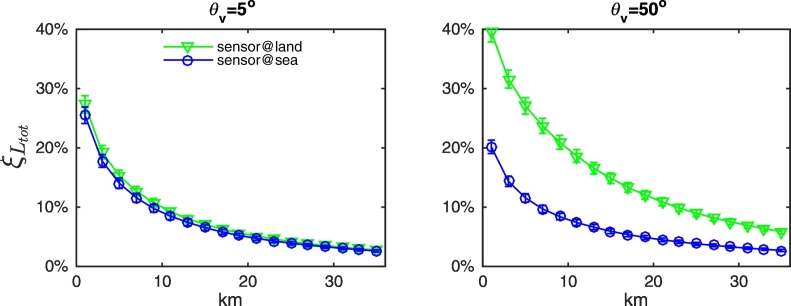
Values of *ξ*_*L*_*tot*__ at 443 nm along the study transect as a function of the distance from the coast for NADR waters in the presence of snow. Observations are for ϕ_0_ = 160° and land located in the western half-plane, while the satellite viewing angle is (left panel) θ_v_ = 5° and (right panel) θ_v_ = 50°. Error bars represent uncertainties. (For interpretation of the references to color in this figure legend, the reader is referred to the web version of this article.)

Results from Fig. 8, Fig. 9, Fig. 10 allow concluding that actual adjacency contributions are expected to be below the average values illustrated in Fig. 7 for quasi-nadir observations and when the sensor is located over the sea. Conversely, they likely are above the average values for slanted observations and when the sensor is located over the land.

It is further remarked that adjacency perturbations are expected to become much more dependent on the sun-sensor position for a coast oriented in the East-West direction.

**Fig. 11:**
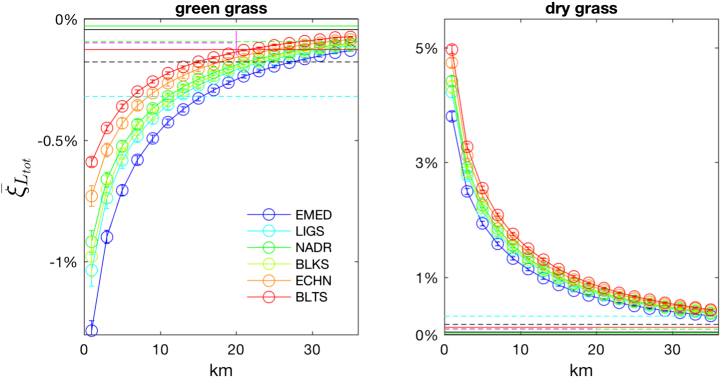
Values of ${\bar{\xi }}_{L_{\mathrm{tot}}}$ at 443 nm along the study transect as a function of the distance from the coast for selected waters and for (left panel) green grass and (right panel) dry grass. Horizontal lines indicate sensors NL (see Fig. 7 for color notations). Error bars indicate uncertainties.

Adjacency perturbations show a slight sensitivity to the water type at the sole blue wavelengths. Specifically, when varying the water type the largest relative changes in the adjacency contributions occur in the presence of poorly reflecting land covers, such as green vegetation or bare soil. In this case, AE decrease with the water optical complexity and are the largest for oligotrophic EMED waters (see the left panel of Fig. 11 for green grass). Conversely, the largest absolute changes occur in the presence of highly reflecting land covers, such as snow, dry vegetation, concrete and white sand. In these cases AE increase with water absorption and are the largest for the BLTS waters (see the right panel of Fig. 11 for dry grass).

Simulations have been performed assuming a constant remote sensing reflectance along the transect. In agreement with results presented and discussed above, *ξ*_*L*_*tot*__ is expected to be sensitive to spatial variations of the water optical complexity at sole blue wavelengths. Results from Fig. 11 suggest that an increase of *R*_*rs*_ towards the coast would lead to a slight increase of adjacency contributions (as compared to Fig. 7) in the presence of green vegetation and bare soil. A slight decrease of adjacency contributions would instead be expected in the presence of more reflecting land covers.

Average adjacency contributions quantified for selected land covers and water types are also summarized in Table 5, Table 6, Table 7 at several distances from the coast and for selected center-wavelengths.

**Table 5:** Values of ${\bar{\xi }}_{L_{\mathrm{tot}}}$ ± 1 standard deviation (in percent) at 443, 670 and 865 nm for selected land covers and NADR waters at 5, 15 and 25 km from the coast. (N = 24 test cases).

	443 nm			670 nm			865 nm		
Land cover	5 km	15 km	25 km	5 km	15 km	25 km	5 km	15 km	25 km
Conifer	− 0.4 ± 0.2	− 0.2 ± 0.1	− 0.1 ± 0.0	− 0.1 ± 0.2	− 0.1 ± 0.1	+ 0.0 ± 0.1	+ 8.9 ± 1.8	+ 3.8 ± 0.9	+ 2.3 ± 0.5
Deciduous	− 0.2 ± 0.2	− 0.1 ± 0.1	− 0.1 ± 0.0	+ 0.0 ± 0.2	+ 0.0 ± 0.1	+ 0.0 ± 0.1	+ 9.5 ± 1.9	+ 4.0 ± 0.9	+ 2.4 ± 0.6
Grass	− 0.5 ± 0.2	− 0.2 ± 0.1	− 0.1 ± 0.1	+ 0.0 ± 0.2	+ 0.0 ± 0.1	+ 0.0 ± 0.1	+ 8.7 ± 1.8	+ 3.6 ± 0.9	+ 2.2 ± 0.5
Dry grass	+ 2.3 ± 0.9	+ 1.0 ± 0.5	+ 0.6 ± 0.2	+ 6.9 ± 1.8	+ 3.0 ± 0.9	+ 1.8 ± 0.5	+ 9.9 ± 2.0	+ 4.2 ± 1.0	+ 2.5 ± 0.6
Medium snow	+ 16.3 ± 4.9	+ 8.1 ± 3.0	+ 4.9 ± 1.8	+ 15.6 ± 3.5	+ 7.2 ± 2.0	+ 4.5 ± 1.2	+ 14.7 ± 2.8	+ 6.5 ± 1.5	+ 3.9 ± 0.9
White sand	+ 8.6 ± 3.0	+ 4.1 ± 1.6	+ 2.4 ± 0.9	+ 12.9 ± 3.0	+5.9 ± 1.7	+ 3.6 ± 1.0	+ 13.6 ± 2.6	+ 5.9 ± 1.4	+ 3.6 ± 0.8
Brown sand	− 0.3 ± 0.2	− 0.2 ± 0.1	− 0.1 ± 0.0	+ 3.2 ± 0.9	+ 1.3 ± 0.4	+ 0.8 ± 0.2	+ 4.7 ± 1.0	+ 1.9 ± 0.5	+ 1.1 ± 0.3
Concrete	+ 3.3 ± 1.3	+ 1.5 ± 0.6	+ 0.9 ± 0.4	+ 5.8 ± 1.5	+ 2.5 ± 0.8	+ 1.5 ± 0.4	+ 6.2 ± 1.3	+ 2.6 ± 0.6	+ 1.5 ± 0.4
Brown loam	− 0.4 ± 0.2	− 0.2 ± 0.1	− 0.1 ± 0.0	+ 2.3 ± 0.7	+ 1.0 ± 0.3	+ 0.6 ± 0.2	+ 4.0 ± 0.9	+ 1.6 ± 0.4	+ 1.0 ± 0.2
Pale brown loam	− 0.2 ± 0.2	− 0.1 ± 0.1	− 0.1 ± 0.0	+ 4.6 ± 1.2	+ 2.0 ± 0.6	+ 1.2 ± 0.3	+ 6.4 ± 1.3	+ 2.6 ± 0.6	+ 1.6 ± 0.4

**Table 6:** Values of ${\bar{\xi }}_{L_{\mathrm{tot}}}$ ± 1 standard deviation (in percent) at 443 nm for selected land covers and EMED, LIGS and BLKS waters at 5, 15 and 25 km from the coast. (N = 24 test cases).

	EMED			LIGS			BLKS		
Land cover	5 km	15 km	25 km	5 km	15 km	25 km	5 km	15 km	25 km
Conifer	− 0.6 ± 0.2	− 0.3 ± 0.1	− 0.2 ± 0.1	− 0.5 ± 0.2	− 0.2 ± 0.1	− 0.1 ± 0.0	− 0.4 ± 0.2	− 0.2 ± 0.1	− 0.1 ± 0.0
Deciduous	− 0.4 ± 0.2	− 0.2 ± 0.1	− 0.1 ± 0.0	− 0.3 ± 0.2	− 0.1 ± 0.1	− 0.1 ± 0.0	− 0.3 ± 0.2	− 0.1 ± 0.1	− 0.1 ± 0.0
Grass	− 0.7 ± 0.2	− 0.3 ± 0.1	− 0.2 ± 0.1	− 0.6 ± 0.2	− 0.3 ± 0.1	− 0.2 ± 0.1	− 0.6 ± 0.2	− 0.3 ± 0.1	− 0.2 ± 0.1
Dry grass	+ 1.9 ± 0.8	+ 0.9 ± 0.4	+ 0.5 ± 0.2	+ 2.2 ± 0.9	+ 1.0 ± 0.4	+ 0.6 ± 0.2	+ 2.2 ± 0.9	+ 1.0 ± 0.4	+ 0.6 ± 0.2
Medium snow	+ 15.4 ± 4.8	+ 7.6 ± 2.9	+ 4.6 ± 1.7	+ 16.0 ± 4.9	+ 7.9 ± 2.9	+ 4.8 ± 1.7	+ 16.1 ± 4.9	+ 8.0 ± 2.9	+ 4.8 ± 1.7
White sand	+ 8.0 ± 2.8	+ 3.8 ± 1.5	+ 2.2 ± 0.9	+ 8.4 ± 2.9	+ 4.0 ± 1.6	+ 2.4 ± 0.9	+ 8.5 ± 2.9	+ 4.0 ± 1.6	+ 2.4 ± 0.9
Brown sand	− 0.5 ± 0.2	− 0.2 ± 0.1	− 0.1 ± 0.1	− 0.4 ± 0.2	− 0.2 ± 0.1	− 0.1 ± 0.0	− 0.3 ± 0.2	− 0.2 ± 0.1	− 0.1 ± 0.0
Concrete	+ 2.9 ± 1.2	+ 1.3 ± 0.6	+ 0.8 ± 0.3	+ 3.2 ± 1.3	+ 1.4 ± 0.6	+ 0.8 ± 0.3	+ 3.2 ± 1.3	+ 1.5 ± 0.6	+ 0.9 ± 0.3
Brown loam	− 0.6 ± 0.2	− 0.3 ± 0.1	− 0.2 ± 0.1	− 0.5 ± 0.2	− 0.2 ± 0.1	− 0.1 ± 0.1	− 0.5 ± 0.2	− 0.2 ± 0.1	− 0.1 ± 0.0
Pale brown loam	− 0.4 ± 0.2	− 0.2 ± 0.1	− 0.1 ± 0.0	− 0.3 ± 0.2	− 0.1 ± 0.1	− 0.1 ± 0.0	− 0.3 ± 0.2	− 0.1 ± 0.1	− 0.1 ± 0.0

**Table 7:** Values of ${\bar{\xi }}_{L_{\mathrm{tot}}}$ ± 1 standard deviation (in percent) at 443 nm for selected land covers and ECHN and BLTS waters at 5, 15 and 25 km from the coast. (N = 24 test cases).

	ECHN			BLTS		
Land cover	5 km	15 km	25 km	5 km	15 km	25 km
Conifer	− 0.3 ± 0.2	− 0.1 ± 0.1	− 0.1 ± 0.0	− 0.2 ± 0.2	− 0.1 ± 0.1	− 0.1 ± 0.0
Deciduous	− 0.1 ± 0.2	− 0.1 ± 0.1	− 0.0 ± 0.0	− 0.0 ± 0.2	− 0.0 ± 0.1	− 0.0 ± 0.0
Grass	− 0.4 ± 0.2	− 0.2 ± 0.1	− 0.1 ± 0.0	− 0.4 ± 0.2	− 0.2 ± 0.1	− 0.1 ± 0.0
Dry grass	+ 2.4 ± 1.0	+ 1.1 ± 0.5	+ 0.6 ± 0.3	+ 2.5 ± 1.0	+ 1.1 ± 0.5	+ 0.7 ± 0.3
Medium snow	+ 16.7 ± 4.9	+ 8.3 ± 3.0	+ 5.0 ± 1.8	+ 17.1 ± 5.0	+ 8.5 ± 3.0	+ 5.2 ± 1.8
White sand	+ 8.9 ± 3.0	+ 4.2 ± 1.6	+ 2.5 ± 0.9	+ 9.2 ± 3.1	+ 4.3 ± 1.7	+ 2.6 ± 1.0
Brown sand	− 0.2 ± 0.2	− 0.1 ± 0.1	− 0.1 ± 0.0	− 0.1 ± 0.2	− 0.1 ± 0.1	− 0.0 ± 0.0
Concrete	+ 3.5 ± 1.4	+ 1.6 ± 0.7	+ 0.9 ± 0.4	+ 3.6 ± 1.4	+ 1.6 ± 0.7	+ 1.0 ± 0.4
Brown loam	− 0.3 ± 0.2	− 0.2 ± 0.1	− 0.1 ± 0.0	− 0.3 ± 0.2	− 0.1 ± 0.1	− 0.1 ± 0.0
Pale brown loam	− 0.1 ± 0.2	− 0.1 ± 0.1	− 0.0 ± 0.0	− 0.1 ± 0.2	− 0.0 ± 0.1	− 0.0 ± 0.0

### 3.2 Benchmark with data from literature

**Fig. 12:**
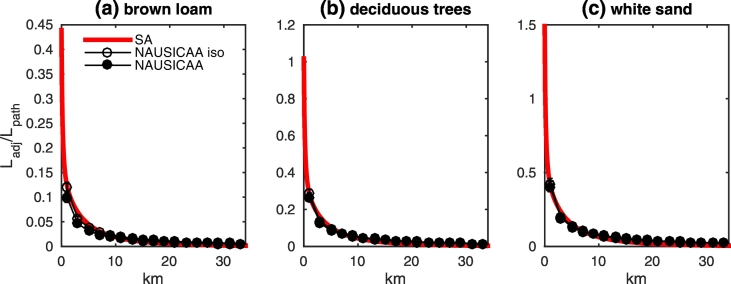
Values of *L*_*adj*_/*L*_*path*_ for nadir observations at 865 nm along the study transect as a function of the distance from the coast for (a) brown loam, (b) deciduous trees, and (c) white sand. The red line indicates *L*_*adj*_ computed with the approximate algorithm by Sei (2007); empty circles represent *L*_*adj*_ simulated with NAUSICAA assuming an isotropic sea surface; full circles also indicate *L*_*adj*_ simulated with NAUSICAA, but fully accounting for the sea surface anisotropy. (For interpretation of the references to color in this figure legend, the reader is referred to the web version of this article.)

Simulated data have been benchmarked with results obtained with the approximate algorithm by Sei (2007) that provides an analytical estimate of *L*_*adj*_ at nadir for two Lambertian half-spaces. For all considered combinations of land and water types, the comparison has been performed for nadir values of *L*_*adj*_/*L*_*path*_, where the path radiance *L*_*path*_, computed with the FEM numerical code (Bulgarelli et al., 1999), describes the radiance due to both atmospheric scattering along the optical path and to specular reflection by the sea surface of the atmospherically scattered light. Results at 865 nm are given in Fig. 12 for a) brown loam, b) deciduous trees, and c) white sand. Values of *L*_*adj*_ from the application of the algorithm by Sei (2007) have been computed by implementing the environment weighting function defined by Reinersman and Carder (1995), but analogous values have been produced when adopting the approximations by Vermote et al. (1997) and by Tanré et al. (1981). Values of *L*_*adj*_ from the application of the NAUSICAA code have been obtained by both assuming a Lambertian sea (in agreement with the assumption adopted in the approximate algorithm by Sei (2007)) and fully accounting for the sea surface anisotropy.

Results show extremely good agreement, particularly in the proximity of the coast where discrepancies between NAUSICAA simulations (fully accounting for the sea surface roughness) and data simulated with the approximate algorithm by Sei (2007) are clearly due to the assumption of an isotropic sea surface in the latter algorithm. The faster convergence with distance from the coast of values simulated with the algorithm by Sei (2007) is the likely consequence of the exponential decay of the environment weighting function. A slower convergence of MC simulations with respect to their exponential approximation was also reported by Reinersman and Carder (1995) (see their Fig. 27).

Analytical results from the algorithm by Sei (2007), extending till the coastal edge, allow evidencing a very steep increase of AE in the first few hundred meters close to the coast where *L*_*adj*_ might reach or even exceed *L*_*path*_ in the presence of a bright land (such as vegetation and white sand, as illustrated in Fig. 12b and c). For the poorly reflecting brown loam, *L*_*adj*_ always remains lower than *L*_*path*_ (Fig. 12a). These results, indicating *L*_*adj*_ exceeding *L*_*path*_ at the coastal edge only, find confirmation in simulations by Santer and Schmechtig (2000). In specific, their *additional reflectance* Δ*ρ*^∗^ (*owing to AE*) for nadir observations over two Lambertian half-planes of albedo *ρ*_l_ = 0.3 and *ρ*_*sea*_ = 0 (see their Table 3) exceed corresponding values of the atmospheric reflectance *ρ*_*atm*_ (see their Table 1) only at zero distance from the coast line, while at 1 km off the coast it is already *Δρ*^∗^ < *ρ*_*atm*_ (corresponding to *L*_*adj*_ < *L*_*path*_). Values of *Δρ*^∗^ exceeding *ρ*_*atm*_ at some distance from the coast are predicted by the same authors only for slanted observations from over the land (see their Fig. 18) and for a dark water disk surrounded by homogenous and infinite land (see their Fig. 2 and Table 2).

Recent empirical estimates of AE determined from MODIS-A NIR data covering clear coastal waters off the Madagascar Island evidenced instead contributions exceeding *L*_*path*_ up to ~ 1.5 km from the coast (Feng and Hu, 2017). The authors of this work noted the difference with AE simulated in the northern Adriatic Sea by Bulgarelli et al. (2014), albeit represented by different statistical indices. Indeed, while both studies assume negligible *R*_*rs*_ at NIR wavelengths (i.e., *L*_*tot*_ = *L*_*path*_ + *L*_*adj*_), the parameter *Ratio* by Feng and Hu (2017) indicates the ratio between the at-sensor radiance from adjacency contaminated and adjacency-free offshore pixels (i.e., *Ratio* = *L*_*tot*_/*L*_*path*_), whereas the parameter *ξ*_*L*_*tot*__ by Bulgarelli et al. (2014) describes the contribution of adjacency radiance at the sensor (i.e., *ξ*_*L*_*tot*__ = *L*_*adj*_/*L*_*tot*_). By accounting for the diversity in the indices adopted in the two approaches, a fully quantitative comparison of AE in the proximity of the coast (~ 1 km) leads to *ξ*_*L*_*tot*__ > 50% in Feng and Hu (2017) and to *ξ*_*L*_*tot*__ ~ 11% in Bulgarelli et al. (2014).

A thorough explanation of these differences would require accounting for the combined effects of averaging over space, uncertainties in the geolocation of satellite data, SNR in the NIR bands, eventual residual NIR contributions from bottom resuspension, anisotropic reflectance of the land surface that may influence AE in the NIR (Bulgarelli et al., 2014), and finally the different observation conditions (i.e., land albedo, atmospheric structure and composition, illumination geometry, and coastal morphology).

**Fig. 13:**
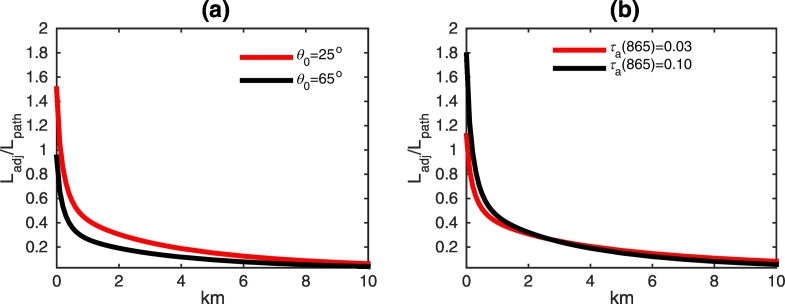
Values of *L*_*adj*_/*L*_*path*_ at nadir for (a) different SZA, and (b) different aerosol optical thicknesses *τ*_*a*_(865) along the study transect as a function of the distance from the coast as computed with the approximate algorithm by Sei (2007) at 865 nm in the presence of white sand. (For interpretation of the references to color in this figure legend, the reader is referred to the web version of this article.)

It is indeed remarked that the eastern coast of Madagascar Island is characterized by a broad leaf non-deciduous forest (IGBP, 2000) and white sand beaches, whose albedos are significantly larger than that of the land facing the northern Adriatic Sea. Further, sun elevation is higher in the tropics than at mid-latitudes, and simulations with the algorithm by Sei (2007) easily show that *L*_*adj*_/*L*_*path*_ tends to decrease with increasing SZA (see Fig. 13a for results at 865 nm in the presence of a white sand land cover) as a consequence of the simultaneous increase of *L*_*path*_ and decrease of *L*_*adj*_ (as evidenced in Bulgarelli et al., 2014). Finally, it is recalled that the horizontal scale of AE (i.e., the distance at which *L*_*adj*_ decreases by a factor *e*) reflects the scale height of the atmosphere (Tanré et al., 1981, Santer and Schmechtig, 2000, Sei, 2007, Reinersman and Carder, 1995), which in turn depends on the load and scale height of the aerosol. Simulations of *L*_*adj*_/*L*_*path*_ at 865 nm, performed with the algorithm by Sei (2007) for H_*a*_ = 1.2 km and for varying aerosol optical thicknesses τ_a_(865), evidence how an increase of aerosol content leads to AE characterized by higher values at the coast and a faster decrease with distance (see Fig. 13b).

It is finally mentioned that the hypothesis by Feng and Hu (2017) that the differences between the two independent quantifications of AE in the NIR at the coastal edge might arise from the assumption of a high sea surface albedo (i.e., 0.04) in the simulations by Bulgarelli et al. (2014) can be definitely excluded. In fact, the methodology applied by Bulgarelli et al. (2014) computes the *Fresnel mask* (i.e., the term ${\tilde{L}}_{\mathrm{ss}}^{\mathrm{TOA}}$ in Eq. (1)) fully accounting for the bi-directional reflectance of a wind roughed sea surface, while parameter *ρ*_*sea*_ (approximated as ~ 0.04 + π*R*_*rs*_ in Bulgarelli et al., 2014) is exclusively used in the determination of the masked water-leaving radiance contribution ${\tilde{L}}_{w}^{\mathrm{TOA}}$ to account for multiple reflections at the sea surface (see Eq. (2)). Notably, when the remote sensing reflectance is negligible (as it is assumed in the NIR), the term ${\tilde{L}}_{w}^{\mathrm{TOA}}$ is null and consequently the parameter *ρ*_*sea*_ does not contribute to any simulated quantity in the NIR.

### 3.3 Biases induced by AE on satellite radiometric products

Adjacency perturbations in satellite radiometric products strictly depend on the AC procedure applied. Estimates of biases induced by AE on the water-leaving radiance derived from satellite data are here presented making reference to the algorithm proposed by Gordon and Wang (1994) and implemented in SeaDAS (Ahmad et al., 2010, Franz et al., 2007), for which the radiance *L*_*tot*_ reaching a space sensor looking at a water element out of the region of sunglint and in the absence of whitecaps is modeled as:

(3) $$L_{\mathrm{tot}}=L_{R}+L_{A}+tL_{w},$$

where *t* is the diffuse atmospheric transmittance (Yang and Gordon, 1997), *L*_*w*_ is the water-leaving radiance, *L*_*R*_ is the radiance resulting from multiple scattering by air molecules in the absence of aerosol, and *L*_*A*_ is the radiance resulting from multiple scattering by aerosol in the presence of air molecules. The latter two terms are intended for an atmosphere bounded by a Fresnel reflecting surface. While the term *L*_*R*_ is assumed exactly known, the aerosol radiance *L*_*A*_ at the visible wavelengths *λ*_*V*_ is derived from the NIR wavelengths *λ*_*N*_ (e.g., 765 and 865 nm, or equivalent), where *L*_*w*_ is assumed a priori known.

Eq. (3) is strictly valid for open-ocean observations, where it is feasible to assume a homogenous underlying water surface. In the vicinity of the coast, Eq. (3) more correctly becomes

(4) $$L_{\mathrm{tot}}=L_{R}+L_{A}+tL_{w}+L_{\mathrm{adj}}.$$

As detailed in Bulgarelli et al. (2017), the bias induced by AE on the derived water-leaving radiance at TOA, *tL*_*w*_, can be expressed as:

(5) $$\psi_{tL_{w}}\left(\lambda_{V}\right)=\frac{L_{\mathrm{adj}}\left(\lambda_{V}\right)}{tL_{w}\left(\lambda_{V}\right)}-\psi_{L_{A}}\cdot \frac{L_{A}\left(\lambda_{V}\right)}{tL_{w}\left(\lambda_{V}\right)}.$$

where *ψ*_*L*_*A*__ represents the bias induced by AE on *L*_*A*_ modeled as:

(6) $$\psi_{L_{A}}=f\left(\;L_{A}\left(\lambda_{N}\right),L_{\mathrm{adj}}\left(\lambda_{N}\right)\right),$$

with *f* representing the function used to infer the aerosol properties from the NIR wavelengths.

Taking this into account, Eq. (5) can be written as:

(7) $$\psi_{tL_{w}}\left(\lambda_{V}\right)=\psi_{tL_{w}}\left[L_{\mathrm{adj}}\left(\lambda_{V}\right)\right]+\psi_{tL_{w}}\left[L_{\mathrm{adj}}\left(\lambda_{N}\right)\right],$$

to indicate the presence of two sources of land perturbations at each wavelength: one induced by AE at the visible wavelength itself, *L*_*adj*_(*λ*_*V*_)/*tL*_*w*_(*λ*_*V*_), and one induced by AE at NIR wavelengths, $-\psi_{L_{A}}\cdot \frac{L_{A}\left(\lambda_{V}\right)}{tL_{w}\left(\lambda_{V}\right)}$ (Bulgarelli et al., 2017).

It is evident from Eq. (7) that when *ξ*_*L*_*tot*__(*λ*_*V*_) < *NL*, the overall bias equals the sole *ψ*_*tL*_*w*__[*L*_*adj*_(*λ*_*N*_)] term. This might for example happen for OLCI-FR radiometric products at 443 nm from ~ 8 km to ~ 10 km offshore in the presence of green vegetation and bare soil, respectively.

In this study, biases are quantified computing *ψ*_*L*_*A*__ in single scattering approximation (see Bulgarelli et al., 2017). It is reminded that this approximation is considered appropriate for an aerosol optical thickness at 865 nm, *τ*_*a*_(865), up to 0.2 (Gordon and Wang, 1994), and it is consequently applicable for the value of 0.06 considered in this study. It is moreover noticed that the single scattering approximation is only applied for the evaluation of *ψ*_*L*_*A*__, while all other terms (*L*_*A*_ included) are computed carefully accounting for multiple scattering.

**Fig. 14:**
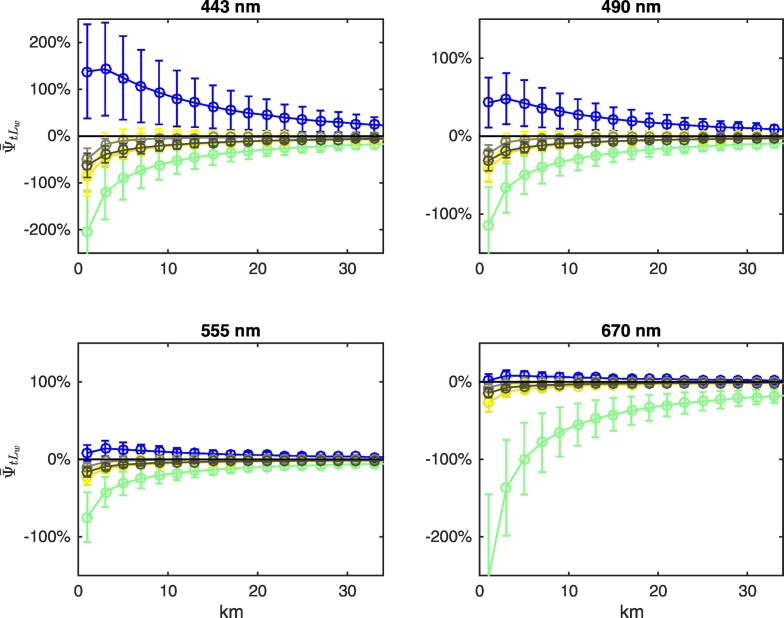
Values of ${\bar{\psi }}_{tL_{w}}$ at representative wavelengths along the study transect as a function of the distance from the coast for NADR waters and representative land covers (see legend of Fig. 7). Error bars indicate ± 1 standard deviation (N = 24 test cases).

Mean biases ${\bar{\psi }}_{tL_{w}}$ are illustrated in Fig. 14 for the same set of cases selected in Fig. 7. Results indicate that for any given wavelength the largest values of ${\bar{\psi }}_{tL_{w}}$ do not necessarily correspond to the largest spectral land albedo. For example, biases for green vegetation are the largest throughout the spectrum, although its albedo at visible wavelengths is the lowest (see Fig. 2). Additionally, although the albedo for dry vegetation is much larger than that of brown loam at all wavelengths, the corresponding values of ${\bar{\psi }}_{tL_{w}}$ are very similar. Furthermore, regardless of the largest spectral albedo of snow (Fig. 2), values of ${\bar{\psi }}_{tL_{w}}$ at 555 and 670 nm for a land covered by snow are comparable to those occurring in the presence of dry vegetation, concrete and brown loam. Notably, the lack of correlation between ${\bar{\psi }}_{tL_{w}}$ and the spectral strength of the land albedo, as well as the non-monotonic spatial trend of ${\bar{\psi }}_{tL_{w}}$ in the proximity of highly reflecting land covers (e.g., snow), show similarities with statistically determined AE in satellite-derived *R*_*rs*_ in the presence of clouds (Feng and Hu, 2016).

**Fig. 15:**
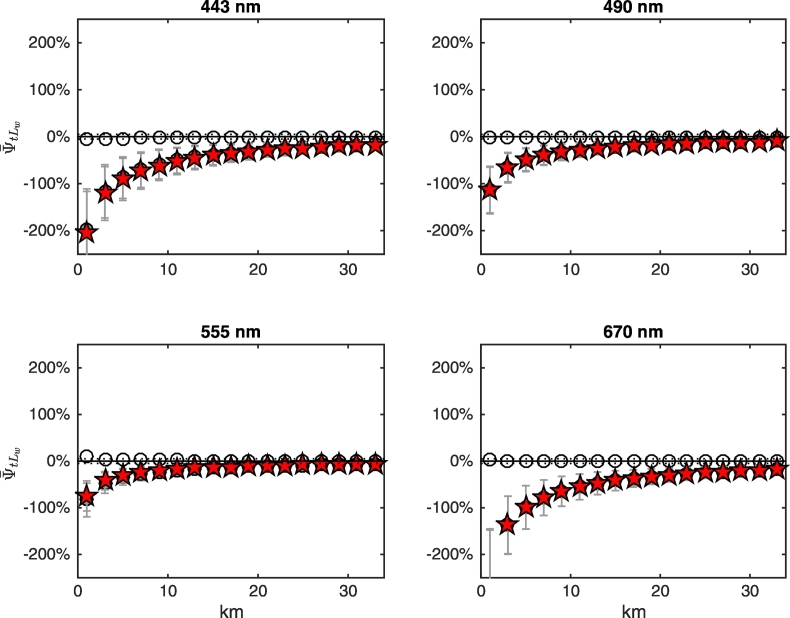
Average spectral values of ${\bar{\psi }}_{tL_{w}}$ (red stars) along the transect together with its components ${\bar{\psi }}_{tL_{w}}\left[L_{\mathrm{adj}}\left(\lambda_{V}\right)\right]$ (white circles) and ${\bar{\psi }}_{tL_{w}}\left[L_{\mathrm{adj}}\left(\lambda_{N}\right)\right]$ (gray circles, here covered by the red stars) for deciduous trees land cover and NADR waters (see Eq. (7)). Error bars represent ± 1 standard deviation (N = 24 test cases). Dotted lines indicate ± 5%. (For interpretation of the references to color in this figure legend, the reader is referred to the web version of this article.)

**Fig. 16:**
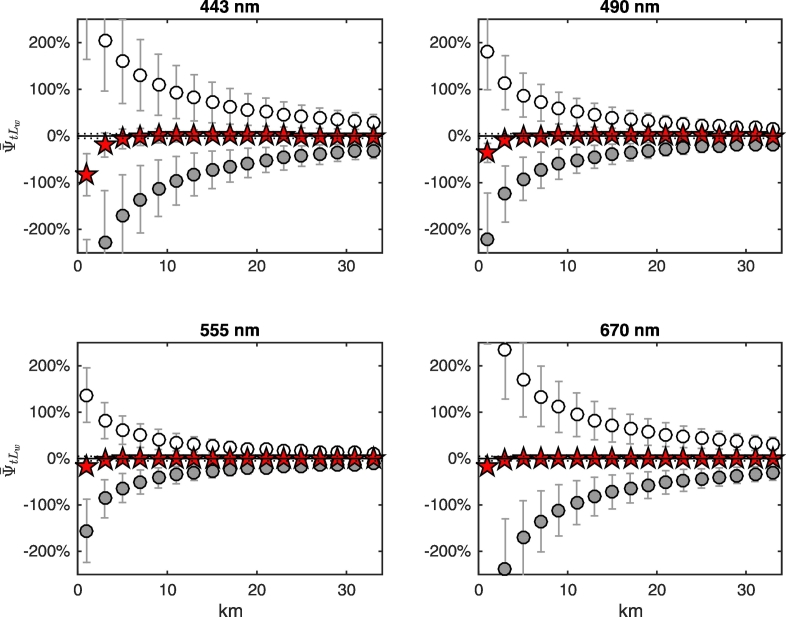
As in Fig. 15 but for white sand.

**Fig. 17:**
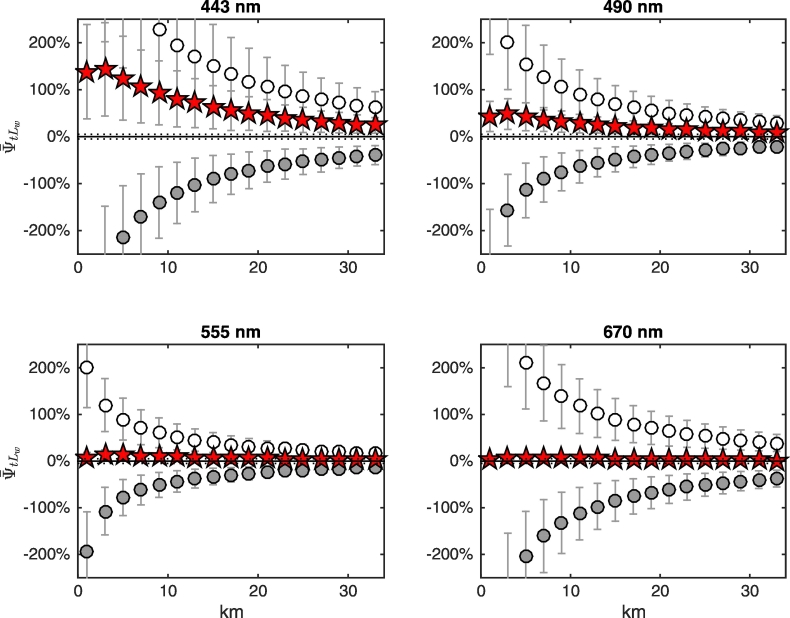
As in Fig. 15 but for snow.

Results illustrated in Fig. 14 are explained by recalling that the two components ${\bar{\psi }}_{tL_{w}}\left[L_{\mathrm{adj}}\left(\lambda_{V}\right)\right]$ and ${\bar{\psi }}_{tL_{w}}\left[L_{\mathrm{adj}}\left(\lambda_{N}\right)\right]$ of Eq. (7) may compensate each other, as illustrated in Fig. 15, Fig. 16, Fig. 17 for NADR waters and deciduous trees, white sand and snow, respectively. For deciduous trees, both components are negative and sum each other leading to values of ${\bar{\psi }}_{tL_{w}}$ up to − 200% at 443 nm at the coast (Fig. 15). For white sand, the positive values of ${\bar{\psi }}_{tL_{w}}\left[L_{\mathrm{adj}}\left(\lambda_{V}\right)\right]$ compensate the negative values of ${\bar{\psi }}_{tL_{w}}\left[L_{\mathrm{adj}}\left(\lambda_{N}\right)\right]$, hence ${\bar{\psi }}_{tL_{w}}$ becomes almost negligible besides in the right proximity of the coast (see Fig. 16). In the presence of snow (see Fig. 17), AE at blue wavelengths are so high that ${\bar{\psi }}_{tL_{w}}\left[L_{\mathrm{adj}}\left(\lambda_{N}\right)\right]$ cannot counterweight ${\bar{\psi }}_{tL_{w}}\left[L_{\mathrm{adj}}\left(\lambda_{V}\right)\right]$ at 443 nm, but compensations still occur at 555 and 670 nm.

**Fig. 18:**
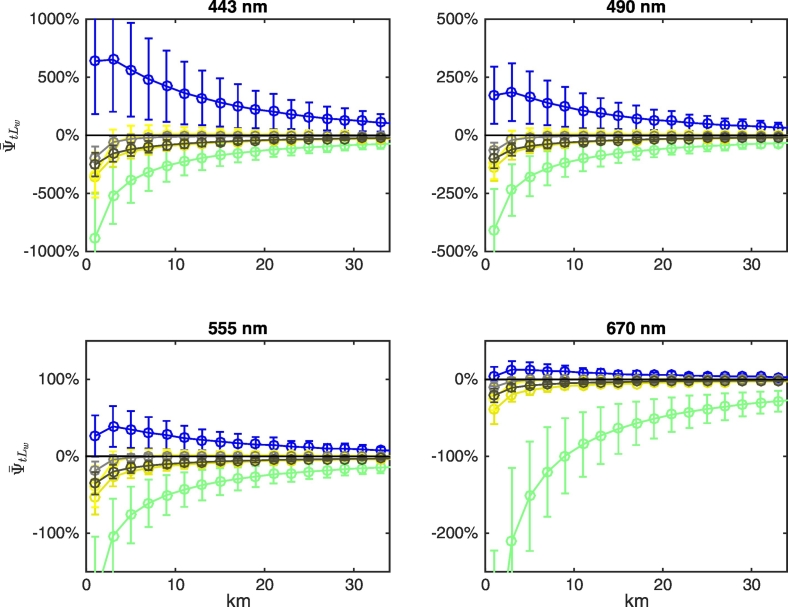
As in Fig. 14 but for BLTS waters.

Biases are chiefly sensitive to the seawater type at blue wavelengths where differences in the water-leaving radiance from different marine regions are the largest (see Fig. 3). Fig. 18 shows biases for CDOM-dominated BLTS waters, whose reflectance at blue wavelengths is very low. In comparison to results in Fig. 14 (for NADR oligotrophic waters), biases at 443 and 490 nm are largely increased (about 4 and 3 times larger, respectively) while they remain almost unchanged at 670 nm.

It is recalled that findings presented so far assume a constant *L*_*w*_ along the whole transect. Following results from Fig. 14, Fig. 18, a decrease of the water signal towards the coast would increase absolute biases. The opposite would occur for increasing *L*_*w*_. Where the land albedo is consistently larger than the sea albedo, the adjacency radiance is not expected to be sensitive to *L*_*w*_. Hence the decrease of biases towards the coast for increasing water turbidity is expected to be proportional to the increase of *L*_*w*_ (Bulgarelli et al., 2017).

Overall results confirm that for an atmospheric correction scheme inferring the aerosol properties from the NIR wavelengths, biases are not directly correlated to the strength of the land spectral albedo. Consequently, the water-leaving radiance determined from satellite data in coastal areas might exhibit larger misestimates for a low reflecting land than for a highly reflecting one.

**Fig. 19:**
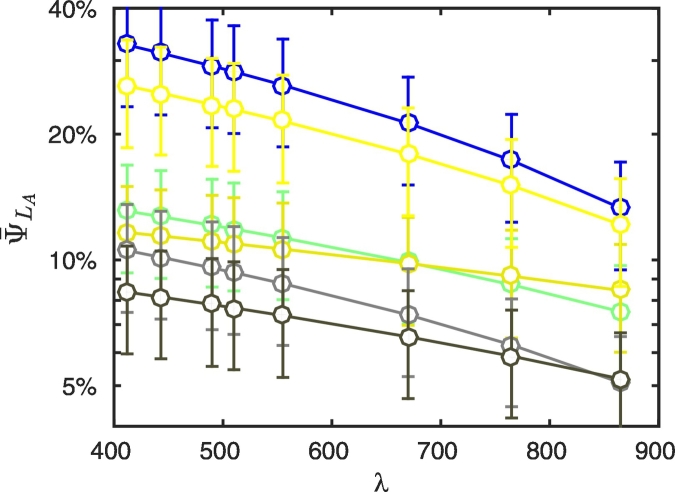
Spectral values of ${\bar{\psi }}_{L_{A}}$ at 9 km from the coast for representative land covers (see legend of Fig. 7). Error bars indicate ± 1 standard deviation (N = 24 test cases).

Finally, Fig. 19 illustrates spectral values of ${\bar{\psi }}_{L_{A}}$ at 9 km from the coast for representative land covers. In agreement with considerations from Bulgarelli et al. (2017), results show that while ${\bar{\psi }}_{L_{A}}\left(865\right)$ is proportional to *ρ*_*l*_(865), larger values of the land albedo in the NIR do not necessarily correspond to larger values of ${\bar{\psi }}_{L_{A}}$ at blue wavelengths. For example, the albedo of concrete in the NIR is about 50% smaller than the albedo of dry vegetation and very close to that of bare soil, nonetheless, ${\bar{\psi }}_{L_{A}}\left(412\right)$ for concrete is very close to that for dry vegetation, while it is significantly larger than that for bare soil. White sand and snow albedos are quite similar in the NIR, nevertheless white sand leads to values of ${\bar{\psi }}_{L_{A}}\left(412\right)$ significantly lower than those occurring for snow. These results find their explanation on *ψ*_*L*_*A*__ that does not only depend on the magnitude of the land albedo at NIR wavelengths, but on its spectral dependence too (Bulgarelli et al., 2017).

It is mentioned that the atmospheric contribution in OLI and MSI data is generally quantified utilizing one or two SWIR bands (Vanhellemont and Ruddick, 2015). As well, SWIR bands can be alternatively used to derive the aerosol optical properties from MODIS data (Wang and Shi, 2007). The analysis of adjacency effects in the SWIR (where the land albedo might consistently differ from values in the NIR) is out of the scope of the present work. Nonetheless, the occurrence of analogous mechanisms of propagation of adjacency perturbations from SWIR to visible, as well as of potential compensations between adjacency perturbations at SWIR and visible wavelengths are expected. A decrease of the impact AE from NIR to SWIR is further envisaged as a consequence of a decrease of atmospheric scattering, sensors SNR (see Table 7 of Hu et al. (2012) for MODIS-A, and Table 2 for OLCI, OLI and MSI), and land albedo (with the exception of bare soil).

## 4 Summary and conclusions

The impact of theoretically estimated AE on SeaWiFS, MODIS-A, MERIS, OLCI, OLI and MSI data has been analyzed under analogous observation conditions accounting for harmonized SNR values. In particular, the SNR of OLCI (in full and reduced resolution), OLI and MSI have been here adjusted to the same input radiance typical of cloud-free ocean scenes as applied by Hu et al. (2012) to standardize the SNR of SeaWiFS, MODIS-A and MERIS. The sensors showing the highest radiometric sensitivity are MERIS-RR at blue center-wavelengths, MODIS-A at yellow center-wavelengths, and OLCI-RR at longer wavelengths. Conversely, SeaWiFS, MERIS-FR and OLCI-FR exhibit lower SNR. MSI and OLI, targeted to land observations, have the lowest radiometric sensitivity, although the radiometric sensitivity of MSI approaches that of MERIS-FR at 443 nm.

Theoretical simulations of TOA adjacency contributions have been performed with the 3D NAUSICAA MC code (Bulgarelli et al., 2014) accounting for multiple scattering within a stratified atmosphere bounded by a non-uniform reflecting surface, sea surface roughness, sun position and off-nadir observation conditions. The simulation exercise has been carried out for a study transect extending up to 36 km (20 km for MSI) from a half-plane of uniform Lambertian land albedo, whose coastline is oriented in the South-North direction. It is recalled that a previous analysis demonstrated the feasibility to adopt a straight coastline without loss in accuracy besides enclosed basins (Bulgarelli et al., 2014). Typical atmospheric conditions and a wide variety of coastal environments have been considered. Selected land covers (whose albedos have been extracted from the ASTER spectral library (Baldridge et al., 2009)) include green and dry vegetation, white and brown sand, snow of different grain size, concrete, bare soils. Water types (whose *R*_*rs*_ have been extracted from the BiOMaP dataset (Zibordi et al., 2011)) span over oligotrophic and mesotrophic waters, and waters dominated by CDOM and/or sediments.

Results highlight that AE from snow, white sand, dry vegetation and concrete are above the sensor noise level NL (equal to 1/SNR) throughout the considered transect, at all wavelengths and for all sensors considered. Conversely, in the presence of bare soil and green vegetation, adjacency contributions might become lower than NL within the study-transect, at a distance increasing with the radiometric sensitivity of the sensor. Finally, while AE in MODIS-A, MERIS, OLCI and SeaWiFS acquisitions at NIR wavelengths are above NL throughout the transect, average adjacency contributions in MSI and OLI measurements due to brown loam are above NL only up to ~ 10 and ~ 26 km, respectively. Where the sea albedo is equivalent or larger than the land albedo, AE are more sensitive to the position of the sun with respect to land. In particular, AE increase with the portion of land included in the solar half-plane. Conversely, when the land albedo is consistently larger than the albedo of the sea, AE become more sensitive to the position of the sensor with respect to the land, being more important when the sensor observe the sea from over the land. AE show a slight sensitivity to the water type exclusively at blue wavelengths, where they evidence an increase with water optical complexity for green vegetation and bare soil, and a decrease for other more reflecting land covers.

Benchmark with results produced with the algorithm by Sei (2007) shows optimal agreement, while further indicates a steep increase of AE in the right proximity of the coast where adjacency contributions might exceed the path radiance in the presence of bright land covers (e.g., snow, vegetation, white sand). Differences with regional empirical estimates of AE from MODIS-A NIR data (Feng and Hu, 2017), may find explanation when accounting for potential uncertainties affecting the quantification of AE in the vicinity of the coast and for differences in the observation conditions, in terms of land albedo, atmospheric structure and composition, illumination geometry and coastal morphology.

For an atmospheric correction scheme deriving the aerosol properties from the NIR wavelengths, the analysis of adjacency perturbations on satellite radiometric products shows that: i) adjacency perturbations on the satellite-derived atmospheric radiance are not only correlated to the strength of the land albedo in the NIR, but depend on its spectral dependence, too; ii) AE in the NIR (affecting the retrieval of the atmospheric radiance) might compensate adjacency perturbations at visible wavelengths, so that even biases on the retrieved water-leaving radiance are not directly correlated to the strength of the land spectral albedo. For example, the impact of AE on the water signal retrieved at blue wavelengths might be larger for a vegetation land cover than for the more highly reflective concrete or white sand. Compensations might even occur in the presence of snow. Over- and underestimates of the radiance for Case-2 moderately sediment-dominated waters (e.g., northern Adriatic Sea waters) at the coast might well exceed ± 100% at 443 nm in the presence of snow and green vegetation, respectively. Misestimates might increase about 4 times for CDOM-dominated waters, like those of the Baltic Sea.

It is mentioned that the retrieval of the atmospheric properties from OLI and MSI data is generally performed utilizing one or two SWIR bands (Vanhellemont and Ruddick, 2015). As well, SWIR bands can be alternatively used to retrieve the aerosol optical properties from MODIS-T and MODIS-A data (Wang and Shi, 2007). Although the analysis of adjacency effects at SWIR wavelengths (where land albedo generally consistently differs from its value in the NIR) is out of the scope of the present work, it is nonetheless expected that analogous mechanisms of propagation of adjacency perturbations from SWIR to visible, as well as potential compensations between adjacency perturbations at SWIR and visible wavelengths, can occur. A decrease of the impact of AE from NIR to SWIR is further expected, as a consequence of a decrease of atmospheric scattering, sensors SNR, and land albedo (with the exception of bare soil).

It is recognized that an actual terrestrial surface is rather a composite of multiple land covers, so that its actual reflectance likely differs from the selected cases. For example, the cropland ecosystem (the most diffuse in Europe) can be modeled as a composite of bare soil and green vegetation, whose reflectance is closer to that of the former in winter and to that of the latter in summer. Nonetheless, the present study offers a useful picture of adjacency perturbations for a number of typical mid-latitude observation conditions of coastal waters from which considerations for specific realistic cases can be drawn.

To summarize, overall results further indicate that the presence of nearby land leads to significant perturbations in remotely sensed data from all the considered sensors. The impact might be limited to the first few kilometers offshore at the sole visible wavelengths and in the presence of green vegetation and/or bare soil. For the highly sensitive MODIS-A, MERIS-RR and OLCI-RR sensors, sole AE at red wavelengths in the presence of green vegetation are expected to be negligible already in the right proximity of the coast. This strongly calls for an operative correction of AE in the new generation of ocean color data addressed to support the exploitation of observations from coastal areas and enclosed waters.

Considering that adjacency contributions at the sensor and even more the perturbations they induce on satellite primary products highly depend on the actual reflectance of land at both visible and NIR wavelengths, an accurate estimate and likely correction of AE relies on the determination of the actual land albedo at the same time and at the same bands utilized for ocean color observations. Future missions targeted to the observation of coastal areas should take this issue into account.
